# Supplementary material for: The effect of M. tuberculosis lineage on clinical phenotype
Source: medRxiv. 2023 Mar 19:2023.03.14.23287284. Preprint. [Version 1] doi: 10.1101/2023.03.14.23287284 (PMC10055556; doi:10.1101/2023.03.14.23287284)
Supplement: 1 [file NIHPP2023.03.14.23287284v1-supplement-1.pdf]

medRxiv preprint doi: https://doi.org/10.1101/2023.03.14.23287284; this version posted March 19, 2023. The copyright holder for this preprint (which was not certified by peer review) is the author/funder, who has granted medRxiv a license to display the preprint in perpetuity.
It is made available under a CC-BY 4.0 International license .

Supplementary Figure 1:

Directed Acyclic Graph (DAG) on the causal assumptions underlying the effect of Lineage on (A) Pulmonary tuberculosis (TB); (B) the presence of Cavity; and (C) Time to culture/smear conversion. Arrows indicate the direction of the effect. Exposure, Mediator, Outcome and Confounders listed below each graph.

medRxiv preprint doi: https://doi.org/10.1101/2023.03.14.23287284; this version posted March 19, 2023. The copyright holder for this preprint (which was not certified by peer review) is the author/funder, who has granted medRxiv a license to display the preprint in perpetuity.
It is made available under a CC-BY 4.0 International license .

Supplementary Figure 2:

medRxiv preprint doi: https://doi.org/10.1101/2023.03.14.23287284; this version posted March 19, 2023. The copyright holder for this preprint (which was not certified by peer review) is the author/funder, who has granted medRxiv a license to display the preprint in perpetuity.
It is made available under a CC-BY 4.0 International license .

Predicted probability for pulmonary tuberculosis (TB) vs. three types of extra-pulmonary TB (TB meningitis, TB osteomyelitis, and other forms of extra-pulmonary TB), by lineage “lin1234” (Lineage 1, 2, 3, and 4), country (Germany and UK) and immigration (“immi” (0 – born local and 1 – born overseas) from multinomial, multivariable regression.

medRxiv preprint doi: https://doi.org/10.1101/2023.03.14.23287284; this version posted March 19, 2023. The copyright holder for this preprint (which was not certified by peer review) is the author/funder, who has granted medRxiv a license to display the preprint in perpetuity.
It is made available under a CC-BY 4.0 International license .

Supplementary Figure 3:

Interval censored regression using proportional hazards models on the association between lineage and time to culture (A) and smear (B) conversion controlling for age, country and immigration. Estimated hazards ratios and bars representing 95% confidence intervals (CIs) are shown on the x-axis. Data from Indonesia, Italy and South Africa all had interval censored data whereas the data from Vietnam were binary (<=60 days or >60 days). The Vietnamese data were therefore converted to interval data (“0 to 60” if <=60; and “61 to ∞ ” if >60). P-values denote evidence of the associations of lineage and time to culture or smear conversion.

medRxiv preprint doi: https://doi.org/10.1101/2023.03.14.23287284; this version posted March 19, 2023. The copyright holder for this preprint (which was not certified by peer review) is the author/funder, who has granted medRxiv a license to display the preprint in perpetuity.
It is made available under a CC-BY 4.0 International license .

Supplementary Figure 4:

Causal mediation analysis (CMA) on the effect of lineage on time to smear conversion mediated by drug resistance and cavity. Estimated time ratios and bars representing 95% confidence intervals (CIs) are shown on the y-axis for each of the decomposition effect including NDE: natural direct effect odds ratio; NIE: natural indirect effect odds ratio; and TE: total effect odds ratio of lineage 2 and lineage 4, compared to lineage 1 as reference. All multivariable models adjusted for country, immigration, and age are shown. P-values denote evidence of natural indirect effect of lineage on time to smear conversion mediated through drug resistance and cavity. The red horizontal lines indicate the thread holds of the results (ORs) of interest.

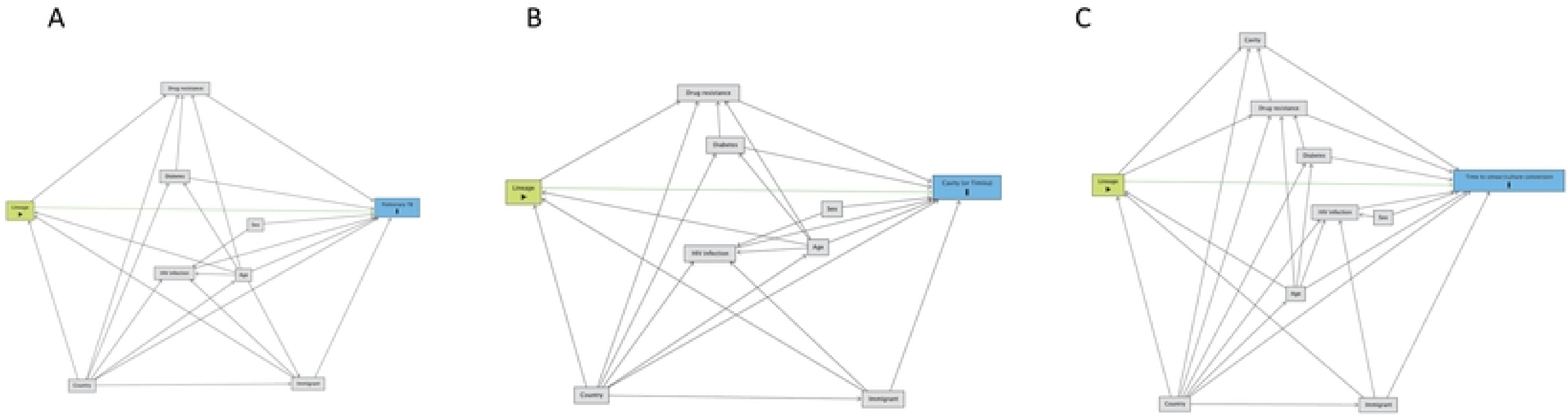

A

Exposure: Lineage
Mediator: Drug resistance
Outcome: Pulmonary TB
Confounders: Country, Immigration, Age

B

Exposure: Lineage
Mediator: Drug resistance
Outcome: Cavity
Confounders: Country, Immigration, Age

C

Exposure: Lineage
Mediator: Drug resistance, Cavity
Outcome: Time to culture/smear conversion
Confounders: Country, Immigration, Age

Supplementary Figure1

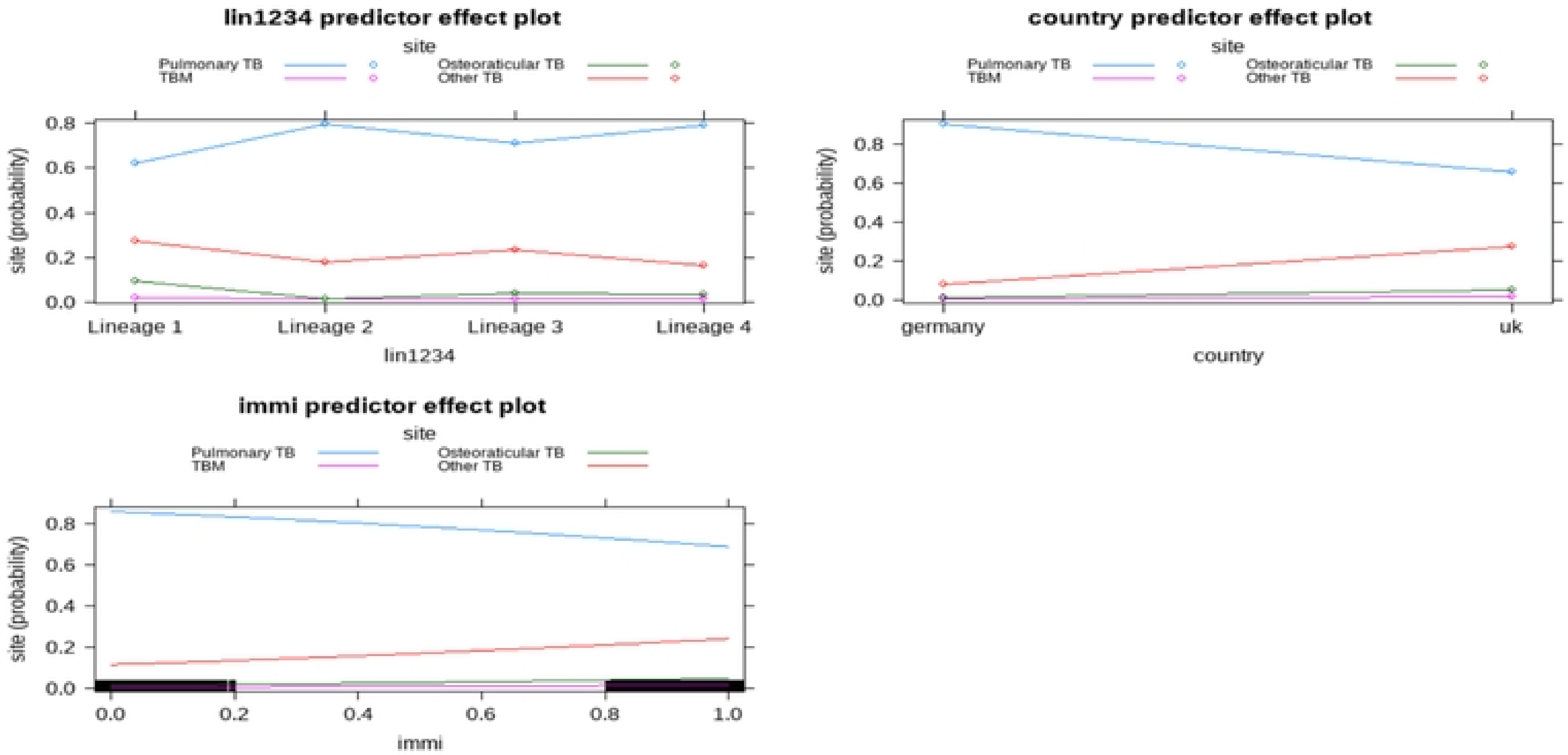

Supplementary Figure2

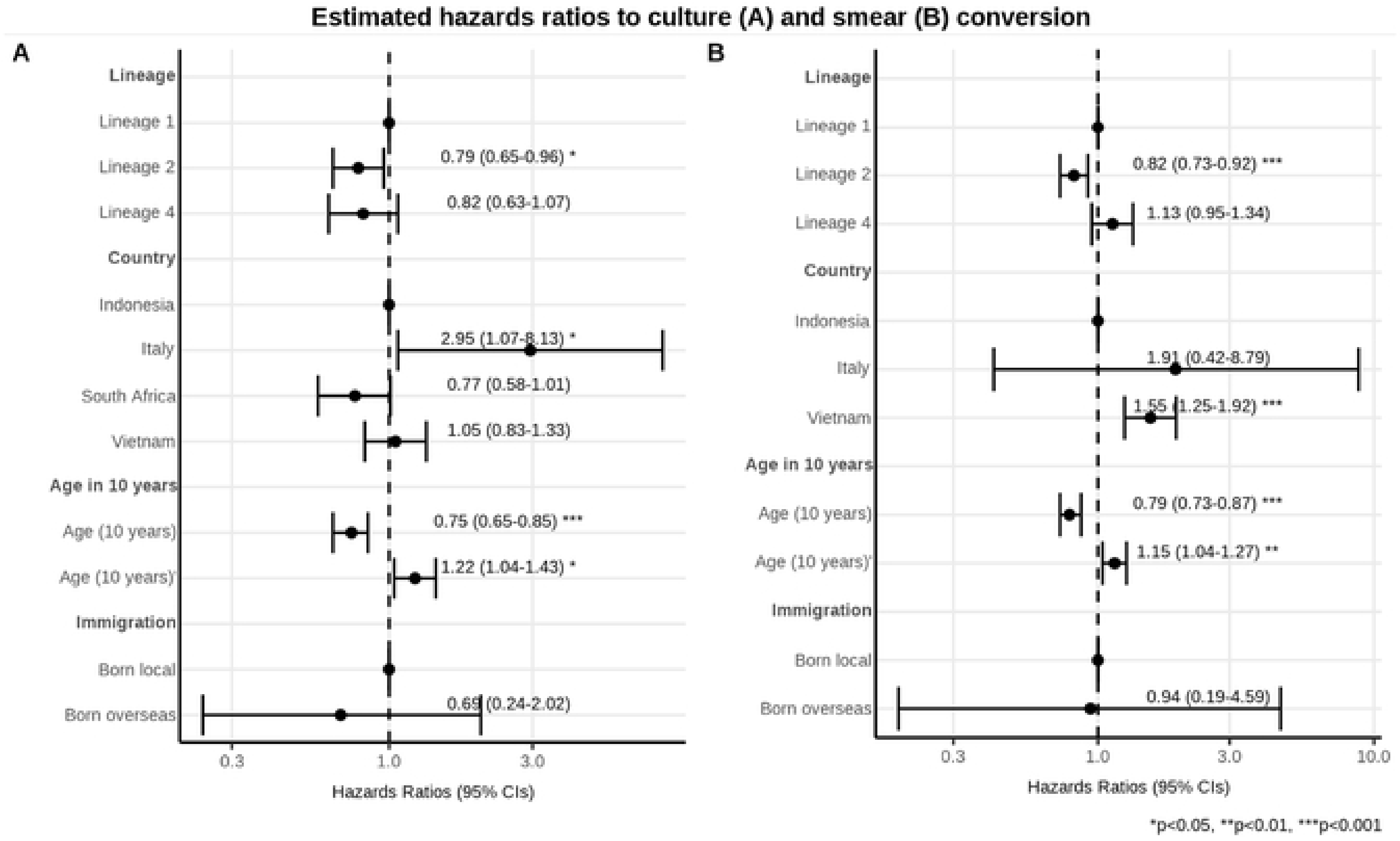

Supplementary Figure3

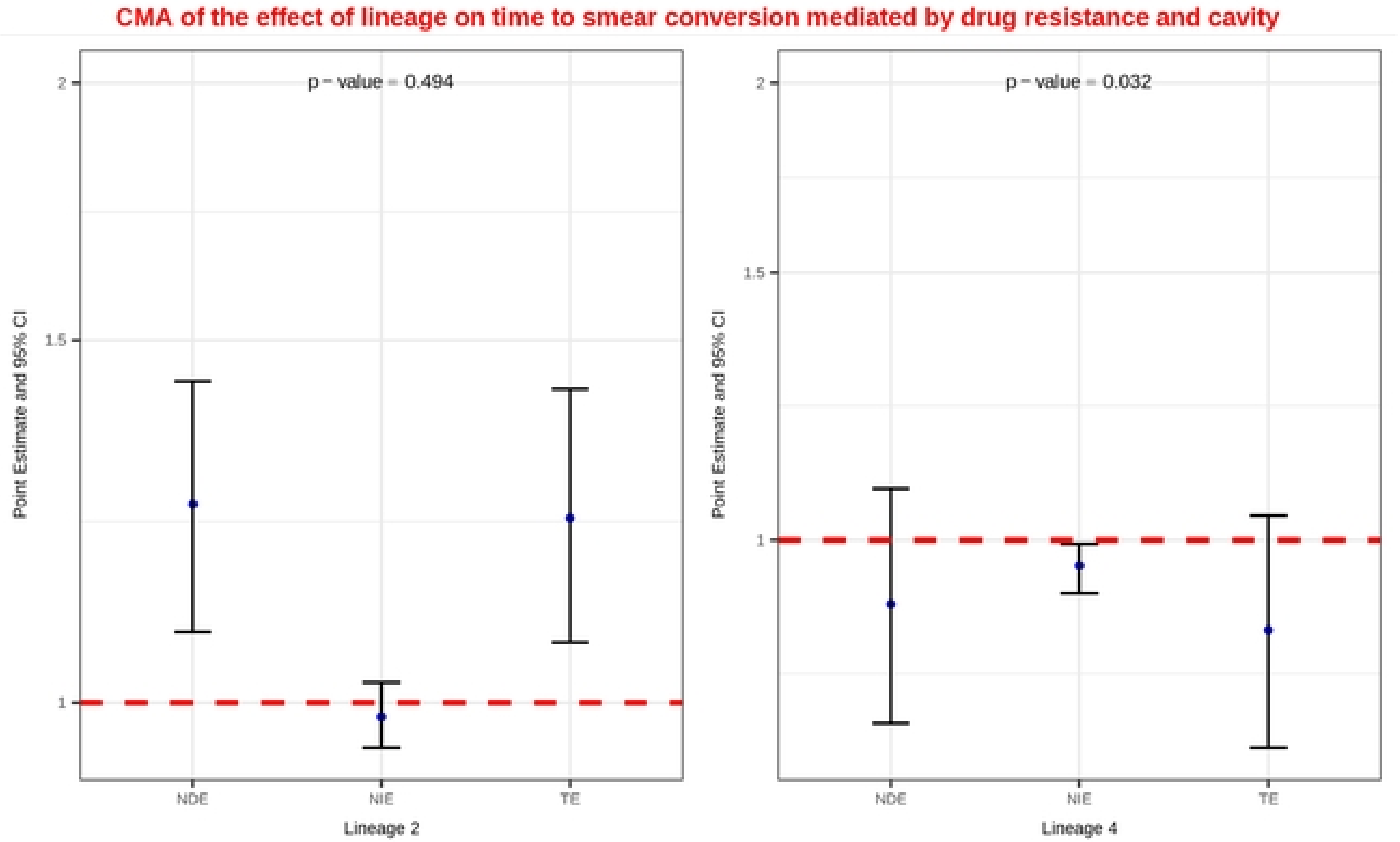

Supplementary Figure4
